# P-2041. Implementing Revised Cefazolin Use Criteria within a Pharmacy-to-Dose Protocol to Improve Guideline-Concordant Antibiotic Prophylaxis in Beta-Lactam Allergic Surgical Patients

**DOI:** 10.1093/ofid/ofaf695.2205

**Published:** 2026-01-11

**Authors:** Cole Orlikowski, Gary Rajah, Ryan Miller, Christopher Ledtke, Jonathan Bott, Dennis Sula, Christopher LaFond, Olivia Juntila, Matthew Satkowiak, Julie Parker, Heidi Davidson, Jessica Maher, Cynthia Nichols, Nicholas Torney

**Affiliations:** Henry Ford Hospital, Detroit, MI; Munson Medical Center, Traverse City, Michigan; Munson Medical Center, Traverse City, Michigan; Munson Medical Center, Traverse City, Michigan; Munson Medical Center, Traverse City, Michigan; Munson Medical Center, Traverse City, Michigan; Munson Medical Center, Traverse City, Michigan; Munson Medical Center, Traverse City, Michigan; Munson Medical Center, Traverse City, Michigan; Munson Medical Center, Traverse City, Michigan; Munson Medical Center, Traverse City, Michigan; Munson Medical Center, Traverse City, Michigan; Munson Medical Center, Traverse City, Michigan; Munson Medical Center, Traverse City, Michigan

## Abstract

**Background:**

β-lactam allergy (BLA) labels in the preoperative setting often lead to avoidance of cefazolin in favor of alternative agents, increasing the risk of surgical site infections, adverse events, antimicrobial resistance, and healthcare costs. Although recent literature supports preoperative cefazolin use in BLA-labeled patients, data on implementing sustainable, scalable system-wide changes to increase cefazolin use remains limited.

**Methods:**

This multi-center, retrospective cohort study included adults with complete allergy data who underwent initial surgery with preoperative antibiotics at one of three Munson Healthcare sites (MMC, MHC, MHG) between 1/1/2020, and 12/31/2024. Outcomes were compared before and after a revision to the preoperative antibiotic prophylaxis guidelines on 2/28/2023, promoting cefazolin use for all patients except those with a cefazolin allergy or a BLA-label consistent with a type II-IV reaction. The primary outcome was rate of prophylactic cefazolin use among BLA-labeled patients, with a subgroup analysis comparing two sites (MMC, MHC) with pharmacy-to-dose (PTD) antibiotic prophylaxis protocols to one site (MHG) without such a protocol. Secondary outcomes included rates of alternative prophylactic antibiotic use and new cefazolin allergies within 24 hours.

**Results:**

Among 39,104 included patients, BLA-label rates were similar pre- and post-intervention (21.0% vs. 20.8%; p=0.56). Following implementation of the revision, cefazolin usage among BLA-labeled patients increased from 69.9% to 90.8% (p< 0.001), while alternative antibiotic use declined from 30.2% to 8.5% (p< 0.001). All sites saw significant increases in cefazolin use among patients with a BLA-label (p< 0.001); however, usage remained higher at PTD sites compared to the non-PTD site both pre- (71.5% vs. 48.1%) and post-implementation (92.6% vs. 65.6%), with a larger absolute increase at PTD sites (p< 0.001). The incidence of new cefazolin allergies within 24 hours remained unchanged (0.01% vs. 0.03%; p=0.26).

**Conclusion:**

Implementing revised cefazolin use criteria within a PTD antimicrobial prophylaxis protocol increased cefazolin use while reducing alternative antibiotic use among BLA-labeled patients, without increasing new cefazolin allergies within 24 hours.

**Disclosures:**

All Authors: No reported disclosures

